# Socioeconomic Interventions for WHO’s End TB Strategy Targets: Insights from SIR Modelling in Kazakhstan

**DOI:** 10.3390/ijerph23030351

**Published:** 2026-03-11

**Authors:** Temirlan Ukubayev, Berik Koichubekov, Marina Sorokina, Donatas Austys

**Affiliations:** 1 Department of Informatics and Biostatistics, Karaganda Medical University, Karaganda 100000, Kazakhstan; koychubekov@qmu.kz (B.K.); m.sorokina@qmu.kz (M.S.); 2 Department of Public Health, Institute of Health Sciences, Faculty of Medicine, Vilnius University, 03101 Vilnius, Lithuania; donatas.austys@mf.vu.lt

**Keywords:** tuberculosis, end TB strategy, socioeconomic interventions, SIR model, forecasting, Kazakhstan

## Abstract

**Highlights:**

**Public health relevance—How does this work relate to a public health issue?**

- This study addresses the persistent public health challenge of tuberculosis in Kazakhstan by modeling its transmission dynamics, directly relating to the global issue of tuberculosis as one of leading causes of death.
- This work highlights how socioeconomic interventions can mitigate transmission risks in middle-income settings, thereby tackling inequities in health outcomes linked to poverty, unemployment, and limited healthcare access.

**Public health significance—Why is this work of significance to public health?**

- The model provides quantitative evidence on how targeted socioeconomic improvements can accelerate tuberculosis incidence reduction, offering a tool for policymakers to prioritize interventions and achieve WHO’s End TB Strategy targets.
- By linking transmission coefficients to socioeconomic predictors and demonstrating robust predictive accuracy, the work advances public health modelling, enabling better forecasting and scenario planning to prevent tuberculosis resurgence amid ongoing global health challenges.

**Public health implications—What are the key implications or messages for practitioners, policy makers and/or researchers in public health?**

- For policymakers, the results emphasize prioritizing financial assistance for TB patients and providing actionable benchmarks to integrate socioeconomic measures into national TB control programs for meeting WHO targets.
- For researchers, the study highlights the value of incorporating socioeconomic factors into dynamic models, suggesting future extensions with drug-resistant strains and stochastic elements to enhance model applicability.

**Abstract:**

Background: Tuberculosis remains a major global public health challenge. Mathematical models are essential for strategic planning and evaluation of tuberculosis control programs, while addressing socioeconomic risk factors has proven key to accelerating incidence declines. Therefore, this study quantitatively assesses the impact of socioeconomic interventions on tuberculosis incidence in Kazakhstan. Methods: A modified SIR compartmental model was developed in Python 3.12 to simulate tuberculosis transmission dynamics. Parameters were calibrated using the Nelder–Mead simplex algorithm, and predictive performance was evaluated via hold-out validation. Scenario-based projections were generated to explore the impact of socioeconomic improvements on future tuberculosis incidence. Results: The calibrated SIR model demonstrated strong predictive accuracy, achieving a mean absolute percentage error of 2.3%. The sensitivity analysis revealed that the model is robust to moderate socioeconomic perturbations, with healthcare funding and unemployment rate as the primary uncertainty drivers. Scenario simulations showed that enhanced financial assistance for tuberculosis patients produced the largest effect beyond baseline. Optimization results indicate that 7.4% rise in GDP per capita, 10.2% increase in healthcare funding, 23.1% and 19.1% reductions in poverty and unemployment rates, and 40.2% growth in tuberculosis patient financial support relative to 2024 are sufficient to achieve the WHO’s End TB Strategy 2030 target. Conclusions: The model offers a valuable tool for tuberculosis forecasting and intervention evaluation, highlighting the synergistic role of socioeconomic measures in achieving global elimination goals.

## 1. Introduction

Tuberculosis (TB) continues to pose a major global public health challenge, despite ongoing efforts by the World Health Organization (WHO) to eliminate it through the End TB Strategy and alignment with the United Nations Sustainable Development Goals. These initiatives aim to substantially reduce TB incidence, mortality rates, and catastrophic costs associated with the disease. Nevertheless, TB remains a significant burden, with an estimated 10.8 million people developing active TB disease in 2023, leading to approximately 1.3 million TB-related deaths [1]. The COVID-19 pandemic has severely disrupted and reversed many years of progress in TB control, severely impacting the delivery of essential TB services and stalling global reductions in TB disease burden. The crisis led to widespread interruptions in routine screening, case detection, diagnosis, treatment initiation, and patient follow-up, resulting in substantial declines in TB case notifications during 2020–2021 and contributing to a significant increase in undiagnosed and untreated cases. Consequently, most of the key global TB targets outlined in the WHO’s End TB Strategy are now off track [2].

TB continues to represent a major public health challenge in the WHO European Region. Kazakhstan is among the high-priority countries of the region, with a TB incidence rate exceeding 30 cases per 100,000 population [3,4]. However, over the past three decades, Kazakhstan has made notable progress in TB control through the nationwide adoption of the Directly Observed Treatment, Short-course (DOTS) strategy in the late 1990s and early 2000s, followed by expanded diagnostics, ambulatory care models, and targeted programs for MDR-TB management after 2005–2006 [5]. These efforts contributed to a sustained decline in incidence and prevalence, although temporary disruptions occurred during the COVID-19 pandemic due to under-reporting and healthcare system strain [6,7].

Mathematical models play a crucial role in the strategic planning and evaluation of TB control programs. They serve as valuable tools for elucidating the transmission dynamics of the epidemic, thereby informing interventions aimed at preventing further spread [8]. In addition, these models enable projections of the epidemic’s future trajectory and facilitate assessments of the potential effectiveness of disease control measures [9].

In 1927, Kermack and McKendrick introduced a deterministic compartmental model to describe the dynamics of epidemic spread, commonly known as the Susceptible–Infected–Recovered (SIR) model [10]. Since its inception, this framework has been extensively adopted and applied to characterize the transmission behavior of numerous infectious diseases [11]. Furthermore, numerous studies have applied the SIR model to forecast future trends in TB incidence and prevalence, as well as to evaluate the potential impact of different control and intervention strategies. These modelling efforts have ranged from estimating the effects of improved treatment coverage and diagnostic speed to assessing the role of vaccination programs, and targeted measures against MDR-TB [12,13,14,15,16,17]. Furthermore, SIR-based models have provided valuable insights into the likely trajectory of the epidemic and the relative effectiveness of public health policies in reducing TB burden over medium–long-term horizons [18,19,20,21,22,23,24].

The realism of transmission dynamics, the accuracy of epidemic projections, and the credibility of intervention evaluations all depend on model parameters—whose precise estimation remains one of the most essential tasks in infectious disease modeling. Among these, the transmission coefficient (β)—which quantifies the rate at which susceptible individuals become infected through contact with infectious cases—is arguably the most influential parameter in determining epidemic trajectories [10,25,26]. In the majority of published compartmental models of TB, β is simply fixed at empirically derived, static values obtained from historical data or literature estimates [27,28,29,30]. This approach, while computationally convenient and often sufficient for short-term descriptive analyses, has several important limitations. It assumes that transmission intensity remains constant over time and may lead to biased forecasts or underestimation of the potential of interventions [31]. Connecting the transmission parameter β to quantifiable socioeconomic indicators provides a more accurate depiction of how structural transformations can alter TB transmission risk. This linkage also establishes a quantitative basis for assessing the epidemiological benefits and returns of implementing social and economic policies.

The current global paradigm for TB control primarily emphasizes interrupting transmission through early case detection and effective treatment, with medical interventions forming the cornerstone of international strategies [32]. Nevertheless, TB has long been recognized as a “social disease”, whose effective control historically required a broader set of social and economic measures [33]. Actions to address socioeconomic risk factors have been demonstrated to play an important role in accelerating the decline in TB incidence and has been incorporated as a central paradigm of the WHO’s End TB Strategy [34]. In this regard, few studies have incorporated socioeconomic variables into dynamic transmission models to explore how social and economic mechanisms influence TB epidemiology and intervention outcomes. Recent study demonstrated that improvements in income and nutrition can significantly reduce tuberculosis transmission and mortality through deterministic compartmental modeling [35]. Another study indicated that investment in preventive interventions can significantly reduce incidence, prevalence, and overall disease burden in high-transmission settings [36]. Furthermore, prioritizing TB control interventions on impoverished populations offers an epidemiological advantage by more efficiently lowering overall transmission and disease burden [37]. Such applications have proven particularly useful in high-burden and middle-income settings, where complex interactions between biological, demographic, and socioeconomic factors shape TB epidemiology. However, the quantitative impact of socioeconomic interventions on tuberculosis incidence remains insufficiently examined receiving limited attention in the existing literature on modeling TB transmission.

Furthermore, the existing literature provides limited guidance on the specific, quantifiable socioeconomic measures that policymakers should prioritize and to what extent they should be pursued to meet the WHO’s End TB Strategy targets. Most studies have focused on descriptive associations, correlational analyses, or qualitative discussions of their importance rather than offering precise estimates of the magnitude and combination of improvements required to achieve the global milestones [38]. This gap is particularly relevant in middle-income settings like Kazakhstan, where TB control has made substantial progress through biomedical and programmatic interventions, yet persistent socioeconomic risk factors continue to sustain transmission at levels that could threaten the achievement of long-term elimination targets [5]. The lack of such evidence limits the ability of national programs to integrate socioeconomic interventions into strategic planning in a targeted manner to bring incidence trajectories in line with the End TB targets.

Therefore, the aim of this study was to quantitatively evaluate the impact of socioeconomic interventions on TB incidence in Kazakhstan through the application of a compartmental model and identify the minimum improvements in key socioeconomic indicators needed to achieve the WHO milestones by 2030 and 2035.

## 2. Materials and Methods

To accomplish the aim of this study, the research proceeded through the following structured steps: (1) development of a modified SIR compartmental model incorporating recurrences, TB-specific mortality, and demographic dynamics; (2) empirical estimation and calibration of key model parameters; (3) assessment of the model’s predictive accuracy; (4) implementation of sensitivity analysis; (5) simulation of multiple hypothetical socioeconomic intervention scenarios.

The analytical pipeline of the study proceeded in a sequential and integrated manner. First, historical TB and socioeconomic data were collected and preprocessed. Next, the transmission coefficient β was empirically calculated from epidemiological flows and then modeled as a function of socioeconomic predictors through principal component analysis (PCA) and quadratic regression. Model parameters γ and ρ were then calibrated using the Nelder–Mead optimization algorithm. Afterwards, model validation was performed via hold-out testing on the 2020–2024 period. Subsequently, the calibrated model was used to simulate a baseline scenario and five target scenarios with annual changes in individual predictors. Sensitivity analysis was conducted using Sobol’s indices to quantify parameter influence and interactions. Finally, inverse optimization was applied to identify the minimum socioeconomic changes required to meet WHO’s End TB Strategy targets for 2030 and 2035.

### 2.1. Model of Tuberculosis Transmission

A modified compartmental SIR model was developed to describe the dynamics of TB transmission in the population of Kazakhstan. The model incorporates recurrent TB cases, TB-specific mortality, and demographic changes. The population is divided into three compartments: S—susceptible individuals; I—individuals with active tuberculosis; R—recovered individuals. Initially, the entire population is assumed to belong to the susceptible compartment (S), implying the same level of immunity and equal risk of infection. Upon the introduction of an infectious individual into this population, transmission occurs, transferring newly infected individuals to the infected compartment (I). Following successful treatment, these individuals are transferred to the recovered compartment (R). Due to the chronic nature of TB, recovered individuals retain a risk of recurrent TB episode and may transition back to the infected compartment (I). Transitions between compartments are governed by specific parameters and rates of change (Table 1).

The recovered compartment, R, is defined biologically and epidemiologically as individuals who have completed treatment and achieved bacteriological cure (negative sputum smear or culture), but who may still harbor latent TB infection or remain at risk of relapse due to incomplete sterilizing immunity. R does not explicitly represent the entire pool of latently infected individuals in the population; rather, it captures those who were previously active cases and have been successfully treated to the point of non-infectiousness.

Regarding recurrence rate, the transition ρR → I is interpreted biologically as relapse (endogenous reactivation of latent infection in previously treated individuals) or, to a lesser extent, treatment failure leading to persistent or recurrent active disease. This flow does not represent exogenous reinfection, which is captured in the transmission flow βSI/N from S to I.

**Table 1:** SIR model parameters and states.

Parameter/ Initial State	Description	Value	Source
N	Population size	15,334,405	Data [39]
S(0)	Initial number of susceptible individuals	15,283,280	Calculated
I(0)	Initial number of infected individuals	51,125	Data [40]
R(0)	Initial number of recovered individuals	0	Assumed
ΔN	The rate of net population growth	−262,765	Data [39]
D	The number of TB-induced deaths	5781	Data [40]
β	The transmission rate	Figure 1	Calculated
ρ	The rate of recurrence	0.350 (1998–2005) 0.105 (2006–2024)	Calculated
γ	The rate of recovery	1.39 (1998–2005) 3.98 (2006–2024)	Calculated

The model is formulated as the following system of ordinary differential equations:

(1) $$\frac{dS}{dt}=\Delta N+D-\frac{\beta SI}{N}$$

(2) $$\frac{dI}{dt}=\rho \;R+\frac{\beta SI}{N}-D-\gamma \;I$$

(3) $$\frac{dR}{dt}=\gamma \;I-\rho \;R$$

where:

*ΔN* is the rate of net population growth;

*D* is the number of TB-specific deaths;

*β* is the rate of infection;

*γ* is the rate of recovery;

*ρ* is the rate of recurrence.

It should be noted that *ΔN* represents annual net population growth, encompassing all demographic changes except TB-specific deaths (*D*). Furthermore, TB-specific deaths are subtracted from the infected compartment (*I*) but added back to the susceptible compartment (*S*) to ensure precise conservation of the total population size *N*(*t*), consistent with observed demographic data. The total population size is defined as:

(4) $$N=S\left(t\right)+I\left(t\right)+R\left(t\right)$$

The construction of SIR model, including parameter calibration, model validation, as well as forecasting, were performed using Python, version 3.12 with packages for data fitting and optimization.

### 2.2. Data Collection

The annual data on TB epidemiology and socioeconomic status of the population for the period from 1997 through 2024 was obtained from the National Scientific Center of Phthisiology and Pulmonology and the Bureau of National Statistics, respectively [39,40]. TB epidemiology was presented by the number of prevalent, incident and recurrent TB cases, TB mortality and successful treatment rates. While socioeconomic indicators were presented by Gross Domestic Product (GDP) per capita (USD), Current Health Expenditure (CHE) as % of GDP, poverty (%) and unemployment levels (%), financial support for TB patients (KZT 1 million).

The years 2020 and 2021 were marked by a significant reduction in notified tuberculosis cases globally and in Kazakhstan, primarily due to under-reporting caused by the COVID-19 pandemic. This reduction resulted from reallocation of healthcare resources, restricted access to diagnostic services, and decreased routine screening activities. According to the WHO, to account for this under-reporting, the incidence data for 2020 and 2021 were adjusted upward by 25% and 10%, respectively [2]. These adjustment factors were derived from country-level analyses and expert consensus to reconstruct the true epidemiological situation and were applied uniformly to ensure consistency with WHO’s global correction approach. The adjustments were made prior to model calibration and were not modified to improve fitting. Therefore, the adjusted incidence values went up from 6696 and 6821 to 8370 and 7500 in 2020 and 2021, respectively. Data for other years remained unchanged, as notification systems had returned to pre-pandemic levels by 2022. All model calibration, validation, and forecasting procedures were performed using this adjusted incidence series to ensure a more accurate representation of the underlying epidemiological trends.

### 2.3. Estimation of Transmission Coefficient β

The transmission coefficient *β* was empirically estimated for each year using observed data on the number of prevalent, infectious and susceptible individuals, as well as the population size:

(5) $$\beta_{t}=\frac{Prevalence(t)N(t)}{Infectious(t)S(t)}$$

To avoid data leakage during hold-out validation, *β* values for the period 2020–2024 were predicted using a quadratic approximation fitted to the observed data. The resulting function was used to extrapolate *β* values forward in time, providing a conservative projection of transmission dynamics.

(6) $$\beta \left(t\right)=at^{2}+bt+c$$

where t=year−1998.

### 2.4. Incorporation of Socioeconomic Factors into the Model

For forecasting purposes (2025–2035), the transmission coefficient β was modelled as a function of socioeconomic factors using quadratic polynomial regression. Annual values of β served as the dependent variable, while socioeconomic indicators (GDP per capita, CHE, poverty and unemployment rates, financial support for TB patients) were included as predictor variables. A quadratic polynomial regression model was fitted to capture potential nonlinear relationships between socioeconomic characteristics and transmission coefficient β.

To mitigate multicollinearity among the socioeconomic predictors, all variables identified during the initial screening were subjected to principal component analysis (PCA) with direct oblimin rotation. Prior to extraction, data adequacy was evaluated using the Kaiser–Meyer–Olkin (KMO) measure of sampling adequacy and Bartlett’s test of sphericity. Eigenvalues for each component were inspected, and the number of factors retained was determined via the scree plot (Elbow criterion), supplemented by retention of components whose eigenvalues exceeded 1.0 according to Kaiser’s rule.

Principal components obtained from the PCA were subsequently used as predictors in a quadratic polynomial regression model to estimate the transmission coefficient β. This step enabled the construction of a predictive equation linking socioeconomic conditions to transmission intensity, while avoiding instability due to multicollinearity among the original predictors. Regression coefficients were estimated using ordinary least squares, and the final model was selected based on goodness-of-fit metrics (adjusted R^2^, residual diagnostics) and accuracy metrics (MAE, MAPE). The resulting regression equation was then applied to forecast β values for future periods in the simulation.

It should be noted that the transmission coefficient β is treated as a descriptive parameter during empirical calibration derived from epidemiological flows and as an explanatory construct during forecasting modeled as a quadratic function of socioeconomic principal components.

### 2.5. Calibration of Recovery (γ) and Recurrence (ρ) Rates

The parameters γ (recovery rate) and ρ (recurrence rate) were calibrated separately for two periods to account for structural changes in tuberculosis control and treatment efficacy in Kazakhstan: 1998–2005 and 2006–2024. The division into these periods was motivated by the observed epidemiological dynamics in the data. In 1998–2005, the prevalence rate was following an upward trend. From 2006 onward, a sustained decline in the prevalence rate began (Figure 2).

Calibration was performed by minimizing the mean absolute percentage error (MAPE) between simulated and observed TB incidence using the Nelder–Mead simplex algorithm, as implemented in the minimize function of the SciPy library (scipy.optimize).

The objective function was defined as:

(7) $$MAPE=\frac{1}{n}\sum_{t=1}^{n}\left|\frac{{Incidence}_{sim,t}-{Incidence}_{obs,t}}{{Incidence}_{obs,t}}\right|\times 100\%$$

where n is the number of years in the calibration period.

### 2.6. Model Validation

**Figure 1:**
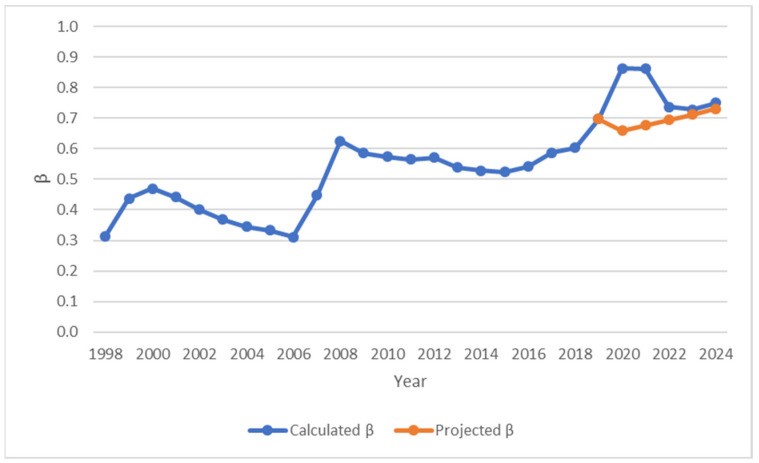
The value of calculated and projected transmission rate β, 1998–2024.

To assess the predictive performance of the model, a hold-out validation approach was employed. The MAPE was calculated as the primary metric. The data were divided into a training set (1998–2019) and a test set (2020–2024) (Figure 1). Predictive performance was evaluated using the MAPE between simulated and observed incidence on the test set.

The quadratic function for the transmission coefficient β was fitted exclusively to the training period (1998–2019) using nonlinear least-squares regression to generate independent predictions of β for the test period (2020–2024).

The parameters γ and ρ were calibrated exclusively on the training set (separately for the periods 1998–2005 and 2006–2019). The resulting parameter values were fixed, and the model was extrapolated to the test period without further optimization.

### 2.7. Sensitivity Analysis

To assess both the individual and joint (interactive) contributions of the predictors to the uncertainty in incidence forecasts, a global sensitivity analysis was performed. Each predictor varied within a range of ±20% from the 2024 baseline value to account for potential crisis conditions or accelerated changes. A sample of 1000 points was generated using the Latin Hypercube Sampling (LHS) method, ensuring uniform coverage of the five-dimensional predictor space. Based on the 1000 resulting incidence values, Sobol’s sensitivity indices were computed: first-order indices (S_i_)—the fraction of variance in incidence explained by each predictor individually; total-order indices (S^t^_i_)—the total contribution of each predictor, including all its interactions with other predictors.

### 2.8. Simulation Scenarios

Long-term projections of TB incidence for the period 2025–2035 were generated using the calibrated parameters from the second period (γ ≈ 3.98 year^−1^, ρ ≈ 0.105).

To explore the potential impact of targeted socioeconomic improvements on future TB dynamics, five scenario-based projections were developed (Table 2). In each scenario, a single socioeconomic predictor was modified annually while all others were held constant at their 2024 values; meanwhile, in the baseline scenario, all the socioeconomic characteristics were fixed at their 2024 empirical value assuming no further changes in transmission intensity.

### 2.9. Optimization of Socioeconomic Predictors

To identify the optimal values of socioeconomic predictors required to meet the WHO’s End TB Strategy targets—an 80% reduction in TB incidence rate by 2030 and a 90% reduction by 2035 compared to the 2015 baseline—an inverse optimization procedure was applied. Optimization was performed using the Nelder–Mead simplex algorithm with initial guesses set near the 2024 baseline values. The resulting optimal predictor values represent illustrative policy guidance needed to meet the WHO targets under the calibrated SIR model dynamics.

### 2.10. Ethical Approval and Considerations

The study was approved by the Bioethics Committee of Karaganda Medical University (Protocol No. 18 on 14 April 2021) with an exemption from informed consent.

## 3. Results

### 3.1. Estimation and Projection of Transmission Coefficient β

Figure 1 presents the calculated annual value of transmission rate *β* for the period 1998–2024.

The results of quadratic approximation of the transmission coefficient *β* produced the following nonlinear least-squares regression:

(8) $$\beta \left(t\right)\;=\;0.000189t^{2}\;+\;0.009067t\;+\;0.3675$$

**Figure 2:**
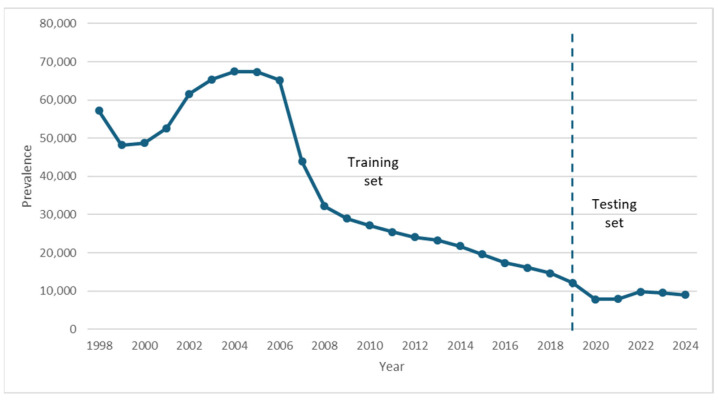
The absolute number of prevalent TB cases from 1998 through 2024 in Kazakhstan.

The nonlinear regression equation projected future values of *β* for the period 2020–2024 (Figure 2). The goodness-of-fit on the training set showed that the residual sum of squares (RSS) was approximately 0.167, the mean absolute error (MAE) was 0.066, and the MAPE was 12.5%. These metrics indicate a reasonable fit, capturing the observed nonlinear trend of increasing transmission intensity over the period. The MAPE for the predicted values of the transmission coefficient *β* on the test period was 11.1% (Appendix A Table A1).

The results of PCA showed that the KMO measure verified the sampling adequacy for the analysis (KMO = 0.682). Bartlett’s test of sphericity indicated that correlations between items were sufficiently large for PCA (x^2^ (10) = 128.5, *p* < 0.001). Two components had eigenvalues over Kaiser’s criterion 1 and in combination explained 89.2% of the variance. Table A2 describes the factor loadings after rotation (Appendix A).

The quadratic polynomial model demonstrated predictive accuracy with MAE of 0.043 and a corresponding MAPE of 6.97%. The results show that the adjusted coefficient of determination (R^2^) was 0.931, implying that the model explained 93.1% of the variance in *β*.

(9) $$\beta \;=\;0.699\;+\;0.003PC1\;+\;0.110PC2\;-\;0.029{PC1}^{2}\;-\;0.014PC1PC2\;-\;0.004{PC2}^{2}$$

### 3.2. Model Fitting and Estimation of Model Parameters

Initial parameter values were set to ρ = 0.05 (corresponding to a 5% annual recurrence probability) and γ = 2.0 (equivalent to an average infectious period of 6 months). Constraints were applied to ensure biological plausibility: ρ ∈ [0, 1] and γ > 0.

**Figure 3:**
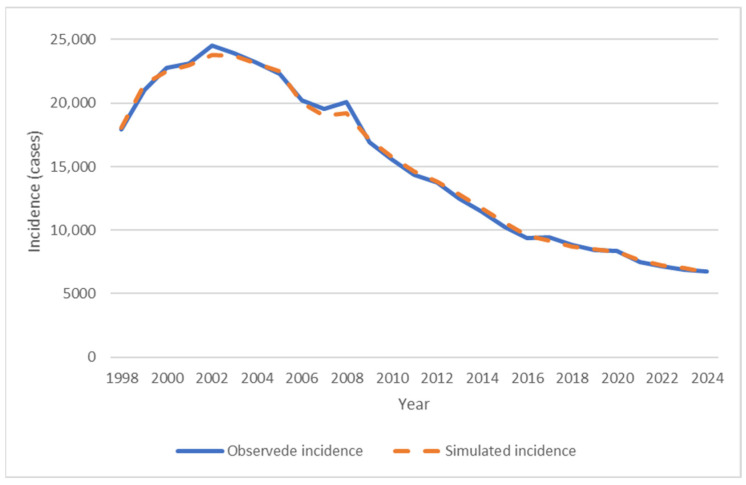
Model fitting to the observed TB incidence.

The results of parameters calibration showed that, for the period 1998–2005, the recovery rate (γ) was estimated at 1.39 year^−1^, corresponding to an average duration of the infectious period of approximately 0.72 years (or about 8.6 months), with a relapse probability (ρ) of 0.35. This period achieved an MAPE of 2.9%. For the subsequent period 2006–2024, the recovery rate increased substantially to 3.98 year^−1^, reflecting a much shorter average infectious period of approximately 0.25 years (or about 3 months); meanwhile, the relapse probability was estimated at 0.105, yielding an MAPE of 9.8%. The overall mean absolute percentage error across the entire period was 7.6%, indicating strong descriptive fit of the model to the observed data (Figure 3).

### 3.3. Model’s Predictive Accuracy

Predictive accuracy was assessed by comparing the simulated incidence with the adjusted observed values. Data for the period 1998–2019 were used for training, while data for 2020–2024 served as the test set.

The parameters γ (recovery rate) and ρ (relapse rate) were calibrated exclusively on the training set, separately for the sub-periods 1998–2005 and 2006–2019. The resulting parameter values were fixed, and the model was extrapolated to the test period without further optimization.

Error analysis revealed a mean value of MAPE of 2.3% for the test period, indicating accurate predictive performance of the model (Table 3).

The transmission coefficient β, annual population growth ΔN, and TB-specific deaths (D) were taken from the actual data for the test period.

### 3.4. Sensitivity Analysis

**Figure 4:**
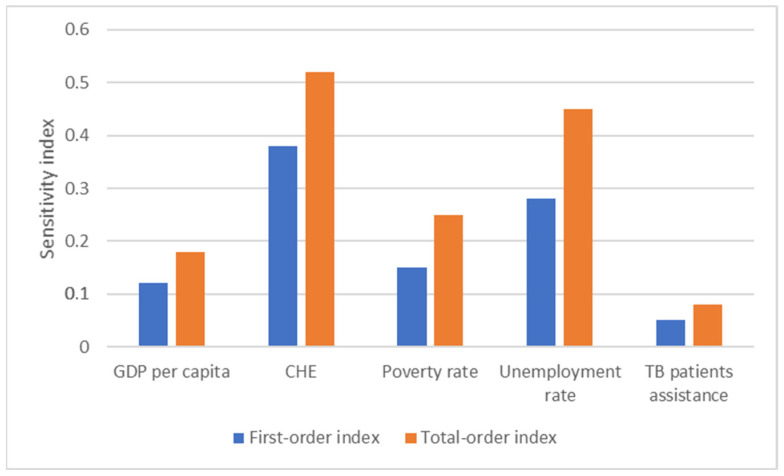
Sobol’s sensitivity indices for TB incidence.

The findings of sensitivity analysis indicate that the model exhibits strong robustness to moderate fluctuations in socioeconomic conditions, with the greatest sources of uncertainty stemming from healthcare funding and unemployment levels (Figure 4).

### 3.5. Simulation Scenarios

Analysis of the simulation scenarios indicated that the annual 3% increase in GDP per capita contributed an additional 1.6–2.1% reduction over the baseline; meanwhile, the annual 0.1 percentage point reduction in poverty and unemployment rates produced only minor extra reductions of 0.9–1.3% and 0.7–1.0%, respectively. Furthermore, the annual 0.1 percentage point increase in current healthcare expenditure (as % of GDP) offered a moderate improvement of 1.8–2.3%. The annual 5% increase in financial assistance for TB patients yielded the greatest extra decline in incidence—between 2.2% and 11.1% beyond the baseline.

### 3.6. Achieving WHO’s End TB Strategy Targets

The optimization results indicate that moderate socioeconomic improvements are sufficient to meet the WHO’s End TB Strategy 2030 target of ≤2052 TB cases (80% reduction). To meet the 2030 target, GDP per capita should rise by at least 7.4% and healthcare funding should increase by at least 10.2% relative to the 2024 baseline levels. At the same time, the poverty rate and unemployment rate need to be reduced by at least 23.1% and 19.1%, respectively, compared to their 2024 baseline values. Additionally, financial assistance for TB patients needs to grow by at least 40.2% from the 2024 baseline.

Achieving the more ambitious 2035 target of ≤1026 cases (90% reduction) demands substantially greater and more sustained efforts. Specifically, GDP per capita should increase by at least 20.1%, while healthcare funding needs to be increased by at least 26.5%. Concurrently, poverty and unemployment rate should be reduced by at least 46.2% and 38.3%, respectively. In addition, financial assistance for TB patients should be increased by at least 100.8% (Table 4).

## 4. Discussion

The results of this study demonstrate that the modified SIR model effectively captures the dynamics of TB transmission in Kazakhstan from 1998 to 2024, providing a robust tool for both historical analysis and future projections. The model, consisting of susceptible (S), infected (I), and recovered (R) compartments, was extended to incorporate recurrent cases, TB-specific mortality, and demographic changes, allowing for a comprehensive representation of the epidemic. Some parameters, such as the transmission coefficient β, were empirically calculated from historical data using the force of infection relationship, while recovery rate (γ) and recurrence rate (ρ) were calibrated through optimization. The model fitting exhibited high accuracy, with an overall MAPE of 7.6%, indicating strong descriptive performance after adjustment for COVID-19 under-reporting. The division into two calibration periods (1998–2005 and 2006–2024) reflected structural shifts in TB control, with the later period showing a substantially higher recovery rate (γ = 3.98 year^−1^) and lower relapse probability (ρ = 0.105), consistent with the nationwide DOTS scale-up and improved treatment regimens.

The hold-out validation further affirmed the model’s predictive capability, achieving an MAPE of 2.3% on the test set. This low error rate, even in a period disrupted by the pandemic, underscores the model’s generalization potential and its ability to forecast incidence trends reliably under real-world conditions.

To incorporate socioeconomic factors, β was represented as a quadratic nonlinear function fitted to the socioeconomic predictors. This modification not only enhanced the model’s realism but also achieved a high degree of validation, with an MAPE of 6.97% for β estimates and 11.1% for projections on the test period.

The sensitivity analysis revealed that the model is robust to moderate variations in socioeconomic conditions, with healthcare funding and unemployment emerging as the primary sources of uncertainty, while interactions among predictors accounted for 60–70% of forecast variance.

The scenario simulations illustrated the potential of socioeconomic interventions to accelerate incidence decline. The annual 5% increase in financial assistance for TB patients was the most effective, yielding an additional 2.2–11.1% reduction beyond the baseline by 2035. GDP growth at 3% per year ranked second (1.6–2.1% additional reduction), followed by the 0.1 percentage point annual increase in healthcare funding (1.8–2.3%). Poverty and unemployment reductions had minor effects (0.9–1.3% and 0.7–1.0%, respectively). Inverse optimization identified minimum socioeconomic changes needed to meet WHO targets, with moderate improvements (e.g., +7.4% in GDP, −23.1% in poverty) sufficient for the 2030 goal and more ambitious efforts (+20.1% in GDP, −46.2% in poverty) required for 2035. These findings emphasize the model’s practical value in quantifying how specific policy levers can bridge gaps to global targets.

From a public health perspective, the model’s high accuracy and robustness offer significant practical applications. The calibration results quantify the impact of Kazakhstan’s TB control advancements, providing evidence that the post-2006 recovery rate improvements have been the primary driver of incidence decline. This supports continued investment in rapid diagnostics, short-course regimens, and adherence support to maintain high recovery rate. The scenario and optimization results provide actionable recommendations for policymakers: prioritizing financial assistance for TB patients and poverty reduction could yield the greatest epidemiological returns, potentially reducing incidence by 10–15% beyond baseline projections. Such interventions, if implemented, could position Kazakhstan to exceed WHO targets, demonstrating the model’s utility in strategic planning and resource allocation. It should be noted that the optimization results are conditional on the model’s simplifications, deterministic dynamics, parameter uncertainty, and the associative nature of socioeconomic relationships.

It is worth mentioning that the adjustments of TB incidence rate in 2020 and 2021 during the COVID-19 pandemic carry inherent uncertainty due to variability in the extent of diagnostic disruptions, notification completeness, and healthcare-seeking behavior across regions and populations during 2020–2021. Alternative assumptions, such as lower correction factors (e.g., 15% for 2020 and 5% for 2021), would slightly increase the overall model MAPE (from 7.6% to 8.2%), while higher factors (35% for 2020 and 15% for 2021) would marginally decrease it (to 7.2%). However, these variations do not meaningfully alter the main parameter estimates, calibration trends, or long-term projections, demonstrating the robustness of the model results to reasonable adjustments in the input data.

Despite the strengths of the model, several limitations must be acknowledged. The assumption of R ≈ 0 in initial conditions, dictated by the data structure where S + I ≈ N, simplifies the model but may underestimate the role of accumulated immunity or latent infection reservoirs. This could lead to overestimation of future susceptibility in the population, particularly in long-term projections where reactivated latent cases might contribute significantly to incidence. Also, the absence of an explicit latent compartment, acknowledged as a deliberate simplifying assumption focused on active transmission dynamics; this may lead to underestimation of long-term reactivation risks in low-transmission settings. Additionally, the single composite transmission coefficient β does not account for subpopulation heterogeneity, such as differences in transmission rates between age groups, or drug-sensitive versus resistant strains. This limitation may bias forecasts in diverse countries, where regional disparities in healthcare access and socioeconomic conditions are pronounced. Furthermore, the deterministic nature of the model omits stochastic variability inherent in real epidemics, potentially leading to overly smooth projections that underestimate uncertainty. The 2020–2021 adjustment for under-reporting, while based on WHO estimates, introduces inherent uncertainty in the magnitude of the correction, as the true extent of missed cases during the pandemic remains imprecise. Finally, the selection of only five socioeconomic predictors limits the model’s scope; additional factors like housing density, migration flows, or nutritional status could provide a more comprehensive representation of transmission drivers, but were excluded due to data availability constraints. Furthermore, the non-causal interpretation of socioeconomic effects on β, which are associative, and derived from historical correlations rather than being strictly causal, with possible confounding, reverse causality, or unmeasured variables not fully accounted for.

It should be noted that the model simplifies the recurrence mechanism by using a single parameter ρ to capture relapse and reactivation among cured individuals, without explicitly distinguishing between endogenous reactivation of latent infection, treatment failure, or exogenous reinfection. This assumption is reasonable for projections focused on active transmission dynamics but may overestimate the contribution of recurrence in settings with high ongoing transmission, where reinfection plays a larger role. Future extensions could incorporate an explicit latent compartment to better separate these processes.

Future research should build on this framework to enhance its precision and applicability. First, extending the model to include stratification for drug-resistant strains would allow for more nuanced projections. Incorporating separate compartments for sensitive and resistant TB, with distinct recovery rates and potentially higher transmission coefficients for resistant strains, could better reflect the challenges of antimicrobial resistance. Second, introducing stochastic elements would account for random variability in transmission and demographic processes. Finally, integrating cost-effectiveness analysis would evaluate the economic feasibility of the identified optimal socioeconomic changes, providing policymakers with comprehensive decision-support tools for prioritizing interventions. These extensions would further strengthen the model’s utility for guiding TB elimination efforts.

## 5. Conclusions

In conclusion, this study offers a robust, integrated framework linking TB epidemiology to socioeconomic drivers in Kazakhstan. The results emphasize that targeted socioeconomic interventions can not only maintain but accelerate progress toward TB elimination, providing evidence-based guidance for national TB control strategies.

## Figures and Tables

**Table 2:** Description of forecasting scenarios for TB incidence (2025–2035).

Scenario	Key Assumption	Source
Baseline	No changes to socioeconomic predictors	Assumed
Sustained economic growth	Annual 3% increase in GDP per capita	Data [41]
Poverty alleviation	Annual 0.1% reduction in poverty rate	Data [41]
Improved labor market conditions	Annual 0.1% reduction in unemployment rate	Data [41]
Enhanced health system investment	Annual 0.1% increase in healthcare funding (as % of GDP)	Assumed
Improved patient support	Annual 5% increase in financial assistance for TB patients	Assumed

**Table 3:** SIR model accuracy.

Year	Observed Incidence (Cases)	Simulated Incidence (Cases)	MAPE (%)
2020	8370	8150	2.6
2021	7500	7720	2.9
2022	7167	7350	2.6
2023	6905	6980	1.1
2024	6733	6650	1.2

**Table 4:** Illustrative policy guidance on socioeconomic predictor values.

Predictor	Baseline	Optimal Value for 2030 Target	Optimal Value for 2035 Target
GDP per capita (USD)	14,155	15,200	17,000
Healthcare funding (% of GDP)	4.9	5.4	6.2
Poverty rate (%)	5.2	4.0	2.8
Unemployment rate (%)	4.7	3.8	2.9
Financial assistance for TB patients (KZT 1 million)	2641	3700	5300

## Data Availability

This published article contains all the data generated or analyzed during the study. The data supporting the findings of this study are available from https://www.gov.kz/memleket/entities/economy?lang=en (accessed on 24 December 2025), https://www.nncf.kz (accessed on 24 December 2025), https://stat.gov.kz/en/ (accessed on 24 December 2025).

## Abbreviations

The following abbreviations are used in this manuscript:

DOTS	Directly Observed Treatment, Short-course
SIR	susceptible-infected-recovered
GDP	Gross Domestic Product
CHE	Current Health Expenditure
KZT	Kazakhstan Tenge
PCA	Principal Component Analysis
KMO	Kaiser–Meyer–Olkin
LHS	Latin Hypercube Sampling
RSS	residual sum of square
MAE	mean absolute error
MAPE	mean absolute percentage error

## Appendix A

**Table A1:** Comparison of forecasted and observed values of the transmission coefficient β during the test period (2020–2024).

Year	Forecasted β	Observed β	Absolute Percentage Error, %
2020	0.659	0.863	23.6
2021	0.676	0.861	21.5
2022	0.694	0.736	5.7
2023	0.712	0.728	2.2
2024	0.731	0.749	2.4

**Table A2:** Factor loadings of the principal components after rotation.

Predictor	PC1	PC2	Communality	Uniqueness
Per capita GDP	0.92	0.12	0.85	0.15
CHE	0.15	0.89	0.81	0.19
Poverty rate	−0.88	0.10	0.78	0.22
Unemployment rate	−0.85	0.14	0.74	0.26
Financial support to TB patients	0.18	0.85	0.76	0.24
